# Ocean acidification effects on mesozooplankton community development: Results from a long-term mesocosm experiment

**DOI:** 10.1371/journal.pone.0175851

**Published:** 2017-04-14

**Authors:** María Algueró-Muñiz, Santiago Alvarez-Fernandez, Peter Thor, Lennart T. Bach, Mario Esposito, Henriette G. Horn, Ursula Ecker, Julia A. F. Langer, Jan Taucher, Arne M. Malzahn, Ulf Riebesell, Maarten Boersma

**Affiliations:** 1 Alfred-Wegener-Institut Helmholtz-Zentrum für Polar- und Meeresforschung, Biologische Anstalt Helgoland, Helgoland, Germany; 2 Norwegian Polar Institute, Framcentre, Tromsø, Norway; 3 GEOMAR Helmholtz Centre for Ocean Research Kiel, Kiel, Germany; 4 National Oceanography Centre (NOC) University of Southampton, Southampton, United Kingdom; 5 Sintef Ocean AS, Marine Resource Technology, Trondheim, Norway; 6 FB2, University of Bremen, Bremen, Germany; University of Connecticut, UNITED STATES

## Abstract

Ocean acidification may affect zooplankton directly by decreasing in pH, as well as indirectly via trophic pathways, where changes in carbon availability or pH effects on primary producers may cascade up the food web thereby altering ecosystem functioning and community composition. Here, we present results from a mesocosm experiment carried out during 113 days in the Gullmar Fjord, Skagerrak coast of Sweden, studying plankton responses to predicted end-of-century *p*CO_2_ levels. We did not observe any *p*CO_2_ effect on the diversity of the mesozooplankton community, but a positive *p*CO_2_ effect on the total mesozooplankton abundance. Furthermore, we observed species-specific sensitivities to *p*CO_2_ in the two major groups in this experiment, copepods and hydromedusae. Also stage-specific *p*CO_2_ sensitivities were detected in copepods, with copepodites being the most responsive stage. Focusing on the most abundant species, *Pseudocalanus acuspes*, we observed that copepodites were significantly more abundant in the high-*p*CO_2_ treatment during most of the experiment, probably fuelled by phytoplankton community responses to high-*p*CO_2_ conditions. Physiological and reproductive output was analysed on *P*. *acuspes* females through two additional laboratory experiments, showing no *p*CO_2_ effect on females’ condition nor on egg hatching. Overall, our results suggest that the Gullmar Fjord mesozooplankton community structure is not expected to change much under realistic end-of-century OA scenarios as used here. However, the positive *p*CO_2_ effect detected on mesozooplankton abundance could potentially affect biomass transfer to higher trophic levels in the future.

## 1 Introduction

Continuous burning of fossils fuels is causing an increase of atmospheric carbon dioxide (CO_2_), and current atmospheric *p*CO_2_ values (ca. 400 μatm) are projected to reach levels of up to 1000 μatm in less than 100 years [1]. Approximately one-third of the anthropogenic CO_2_ has been taken up by the oceans [2] leading to a reduction in pH (hence the term “ocean acidification” [3, 4]) and shifts in seawater carbonate chemistry [5]. Coastal marine ecosystems may be less sensitive to increased CO_2_ than open ocean regions, as the natural CO_2_ fluctuation in these areas is already substantial [1, 6]. However, ocean acidification (OA) can interact with other natural and anthropogenic environmental processes such as warming [7], eutrophication [8], and deoxygenation [9], making it a potential threat in conjunction with other stressors. Furthermore, OA may affect zooplankton not only directly by decreases in pH, but also indirectly via trophic pathways [10–12]. Consequently, both direct pH as well as *p*CO_2_ effects on primary production [13] may travel up the food web [10] therefore altering ecosystem functioning and community composition (e. g. [14]).

Elevated *p*CO_2_ in seawater may have positive effects on primary production, but at the same time impact marine organisms both via changes in calcification rates [15, 16], and via disturbance to acid–base (metabolic) physiology [17]. Calcified secretions in marine fauna and flora are not only limited to skeletal CaCO_3_ (thus, calcifiers *sensu stricto*) but there are other calcium-based structures that might be a target for low pH effects, such as, for example, the equilibrium organs (statoliths) in gelatinous zooplankton [17]. These organs are calcium magnesium phosphate crystals which may be affected by lowering pH [18], as reported for statoliths of scyphomedusae [19].

Copepods are the most abundant marine planktonic metazoans and, together with microzooplankton, are the major primary consumers in most marine food webs, sustaining secondary consumers such as fish and jellyfish [20, 21]. Copepods typically prefer larger and moving prey, i.e. they feed primarily on ciliates and dinoflagellates than on diatoms [22, 23], with preferred sizes between 20 and 200 μm ([24] and the references therein). As a result, they often switch from phytoplankton to microzooplankton over the course of a phytoplankton bloom [22] as larger prey items typically only become available later in the phytoplankton bloom, and even predate their offspring when resources are scarce [25].

Previously, copepods were considered to be relatively tolerant to OA [26, 27], but several processes in copepods may in fact be affected by low pH, including metabolism [28], pH balance [29], reproduction [30], development [31], growth [32] and survival [33]. Furthermore, diverse sensitivities to OA exist between different species and even between life stages within species [34]. Early life stages are most sensitive, resulting in a potential negative effect on survival and/or development (e. g. [29, 30, 35]). Different sensitivities to OA might also be related to copepod habitats, thus those copepod species more exposed to natural pH fluctuations (as vertical migrators or coastal species) might be more tolerant to OA than others [33, 36].

During the last decade, numerous studies dealing with the potential effects of high CO_2_ on single species were published (e. g. [35, 37]), while ecosystem-level impacts have attracted less attention. In order to assess future OA effects on natural communities, studies focused on ecological interactions (e. g. [38–41]), as well as long-term multigenerational experiments [42–44] are of paramount importance. To investigate the effects of end-of-century *p*CO_2_ levels on coastal pelagic ecosystems, we conducted a long-term mesocosm experiment in a boreal fjord. The present paper is part of the BIOACID II long-term mesocosm study PLoS Collection [45]. Here we focus on the natural mesozooplankton community, in particular on copepods and hydromedusae as the most abundant taxa. Testing the null hypothesis of no-effect, we assessed (1) mesozooplankton community development along the winter-to-summer plankton succession and the OA effects on the community interactions as well as (2) temporal trends and high-CO_2_ effects on species abundances, supported by two onshore experiments in the case of the most abundant copepod species, *Pseudocalanus* a*cuspes*.

## 2 Materials & methods

### 2.1 Mesocosms setup and experimental design

**Fig 1:**
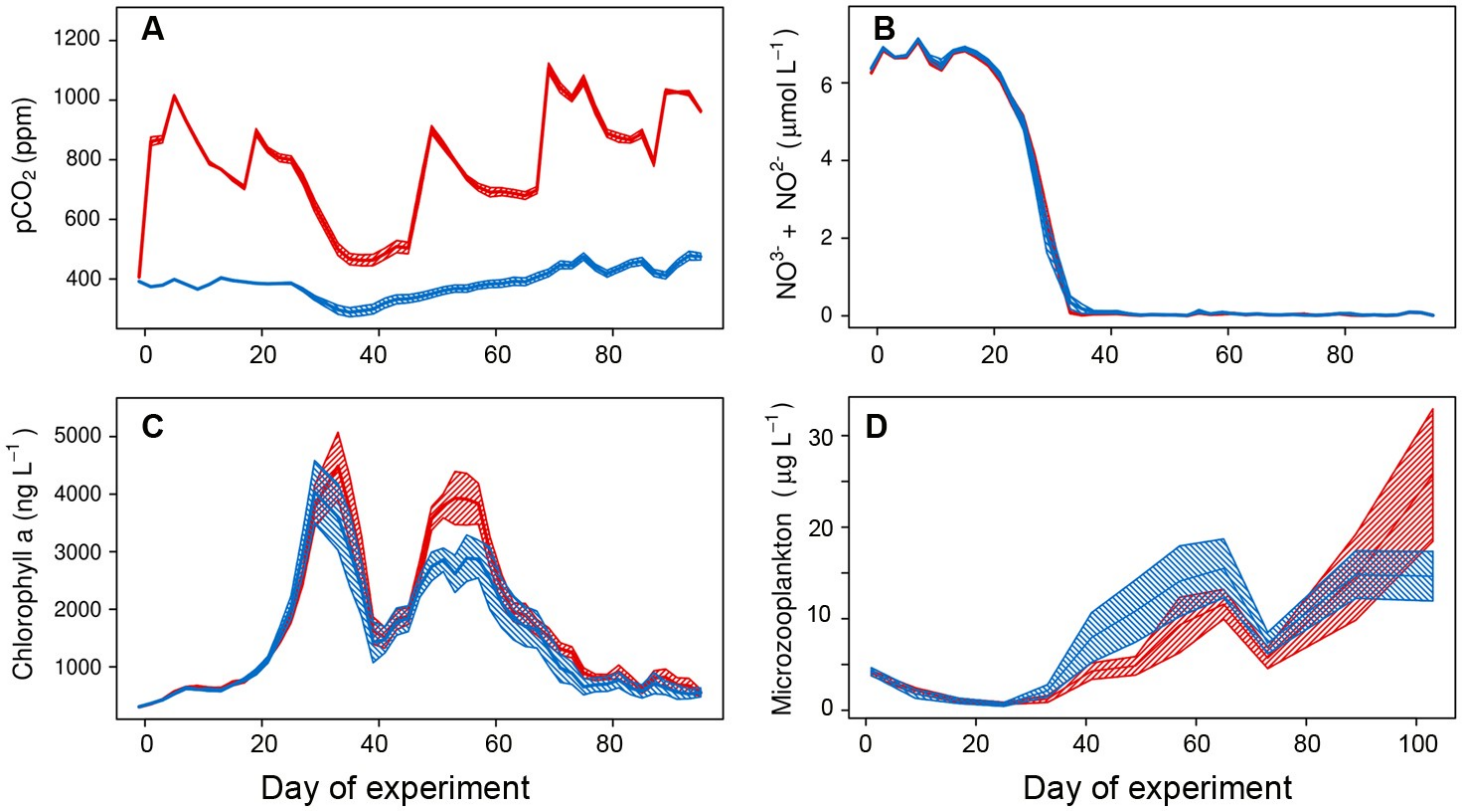
Abiotic and biotic factors potentially affecting mesozooplankton community along the experiment. a) *in situ p*CO_2_ levels, b) nutrients (NO_3_^-^+ NO_2_^-^), c) chlorophyll *a*, and d) microzooplankton abundances (ciliates and heterotrophic dinoflagellates). Colour code: red = treatment (~760 μatm *p*CO_2_), blue = control (ambient conditions). Solid lines = mean values; striped area = standard error of the mean.

Within the framework of the BIOACID II project (Biological Impacts of Ocean ACIDification), this study was part of the”BIOACID II long-term mesocosm study”, which was conducted from January to July 2013 in the Gullmar Fjord (58°15’ N, 11°28’ E), on the Swedish Skagerrak coast [45]. We deployed ten mesocosms (KOSMOS, M1-M10: “Kiel Off-Shore Mesocosms for future Ocean Simulation”, [46, 47]) in the fjord to study the effect of changing carbonate chemistry conditions on mesozooplankton community development. The experimental units consisted of large enclosed water volumes (~50 m^3^), five of them used as controls (ambient *p*CO_2_ levels = ca. 380 μatm), and the other five were CO_2_-enriched in levels adjusted to realistic end-of-century scenarios (RCP 6.0 [1]). Mesocosms were sealed by sediment traps, installed at the bottom of each mesocosm bag. Target *p*CO_2_ was reached at the beginning of the experiment by adding CO_2_ saturated seawater to the mesocosms. Subsequent additions were made on a regular basis in the course of the experiment (day 17, 46, 48, 68 and 88) to compensate for CO_2_ loss through outgassing. We established realistic end-of-century *p*CO_2_ levels (average = ca. 760 μatm) over the study period (see Fig 1a, [45]). Regular sampling every 2^nd^ day included CTD casts, water column sampling, and sediment sampling. Water column samples were collected with integrating water samplers (IWS, Hydrobios), which collect a total volume of 5 L from 0–17 m depth evenly through the water column. This water was used for nutrient analyses, pigment analysis, and microzooplankton microscopy. All analyses are described in detail in [45] within this PLoS Collection. Briefly, nutrient (NO_3_^-^+ NO_2_^-^) concentrations (Fig 1b, [45]) were measured with a SEAL Analytical QuAAtro AutoAnalyzer and a SEAL Analytical XY2 autosampler. Pigment extracts were used for analysis by means of reverse phase high performance liquid chromatography (HPLC) (Fig 1c, [45]). Every eight days, microzooplankton samples were taken from the IWS carboys, immediately fixed with acidic Lugol’s solution and stored dark until identification (Fig 1d, [48]). Results presented here correspond to t_1_ (10^th^ March) up to t_103_ (20^th^ June) of the 113 days that the mesocosms experiment lasted [45].

### 2.2 Mesozooplankton sampling

The mesozooplankton community was sampled in the mesocosms and the fjord by vertical net hauls with an Apstein net (55μm mesh size, 17 cm diameter) equipped with a closed cod end, sampling a total volume of 385 L. Sampling depth was restricted to the upper 17m to avoid resuspension of the material accumulated in the sediment traps, at 20m depth. One net haul per mesocosm was taken once every eight days, within a narrow time-window (1 to 3 p.m.) to avoid differences in the community composition caused by diel vertical migration. Note that sampling frequency was lower than for other water column samples to avoid overharvesting of the plankton community. Samples were rinsed on board with filtered sea-water, collected in containers and brought to the laboratory, where samples were preserved in 4% formaldehyde buffered with sodium tetraborate. For transportation during summer time, the samples were placed in cooling boxes until fixation of the organisms.

During analysis, organisms were sorted using a stereomicroscope (Olympus SZX16) and classified to the lowest possible taxonomical level, including gender in the case of adult copepods. Copepodites and adults were classified to species level whereas nauplii from different species were pooled together. Taxonomical analyses were carried out focusing on copepods [49–52] and hydromedusae [53–55] as the most abundant groups. Every sample was sieved through 50 μm mesh, rinsed with tap water and poured into a calibrated beaker, where organisms were well mixed before taking a 5% aliquot with a Hensen Stempel pipette [56]. Counting was restricted to 5% (one aliquote) or 10% (two aliquots) of the total sample for the most abundant groups (nauplii, *P*. *acuspes* adults and *P*. *acuspes* copepodites) when more than 200 individuals were counted in the first aliquot. Otherwise the subsampling procedure was repeated, counting a maximum of a 15% of the total sample for all species.

Since some organisms characteristic to a winter-to-summer succession might not have been included when the experiment started, the community within the mesocosms was enriched by the addition of 22 L of fjord water every fourth day [45]. Likewise Atlantic herring (*Clupea harengus*) eggs and green sea urchin (*Strongylocentrotus droebachiensis*) gastrulae were artificially added to each mesocosms on t_48_ and t_56_ respectively [45] according to the time of the year that these groups would have been part of the natural fjord community. Densities of herring eggs introduced in the mesocosms were ~70–108 eggs per m^3^ and peak egg-hatching was estimated to occur around t_63_, with a final number of 1608 ± 237 hatched larvae per mesocosms, i. e. ~27–37 larvae per m^3^ [57]. These larval densities are within the natural range for the North Sea [58]. Sea urchin gastrulae were obtained in the onshore laboratory, introduced in the mesocosms (~110 sea urchin gastrulae per m^3^) and subsequently monitored from the mesozooplankton net tows on a weekly basis. An in depth analyses of Atlantic herring and green sea urchin larvae development are provided by Sswat et al. [57] within the framework of this PLoS Collection and Dupont et al. (unpubl. data).

### 2.3 *P*. *acuspes* condition experiments

Copepods were the most abundant group within the mesozooplankton community during the whole experiment, and the calanoid copepod *P*. *acuspes* was the most abundant species. To gain insights in *P*. *acuspes*’ physiological response to simulated OA we conducted two additional incubation experiments during the pre-bloom (March, t_19_) and senescence phase (May, t_59_) of the phytoplankton community (Fig 1). Every mesocosms was sampled by an extra net haul (see 2.2), and *P*. *acuspes* females were sorted immediately and subsequently incubated in a cold room adjusted to the average *in situ* temperature (t_19_: 3°C and t_59_: 5°C [45]) for offspring viability monitoring (*n* = 12) and respiration measurements (*n* = 5), or preserved for carbon content analyses (*n* = 20). Normally swimming females with undamaged eggs (60 females per treatment) were selected and initial clutch sizes were noted prior incubation to assess hatching rates. We aimed to incubate 12 females per mesocosms (i. e., 60 females per treatment), but this was not achieved in all cases due to the scarcity of egg carrying females within some samples or due to mortality of the females after 24h. Considering that incubation in small volumes does not affect egg production [59], females were incubated for 48h in 6-well plates, one female per well, in starvation and simulated field temperature. No additional *p*CO_2_ treatment was necessary because the aim of this side experiment was to analyse the memory effects of increased *p*CO_2_ on females in the mesocosm rather than effects on the eggs themselves. Clutch size and survival of the females were recorded each day during the condition experiments. Prosome length of all incubated females was measured upon termination of the experiment.

Respiration rates of five egg-carrying females per mesocosm (i. e. 25 animals per treatment) were measured in the cold room. Females were transferred to 1.6 mL vials equipped with fluorescent O_2_ foil discs (PSt3 spots, PreSens Precision Sensing, Germany) and filled with seawater adjusted to the *p*CO_2_ levels from corresponding mesocosms, based on the immediately preceding carbonate chemistry measurements in the mesocosms [45]. Vials were then sealed with Teflon caps and O_2_ concentrations were measured at 0, 3, and 6 hours using a Fibox 3 optode system. Respiration rates were calculated by subtracting the average oxygen depletion rate measured in five controls from the oxygen depletion rate in the vials holding copepods, multiplying by vial volume and dividing by number of individuals in each vial. Prior testing of the optode system at 5°C showed a 2 min 95% reaction time, i.e. the period of time taken before the output reached within 5% of the final oxygen concentration value (as estimated by exponential regression). Therefore, at every sampling, oxygen concentrations were read for three minutes, and an average of values read during the last minute was used for calculations.

To analyse carbon content, 20 non-ovigerous *P*. *acuspes* females were sorted from each mesocosm sample (i. e. 100 animals per treatment). The females were briefly rinsed in Milli-Q water to remove the excess of salt, and preserved in pre-weighted tin cups, which were in time dried (60°C) and preserved in an desiccator until analysed. Weights were obtained with a microbalance (Sartorius SC2). A Vario MICRO cube CHN analyser (Elementar) was used to measure carbon content.

### 2.4 Statistical analysis

To study Gullmar Fjord’s mesozooplankton community we firstly calculated species diversity for every mesocosm, which were compared using general linear models (GLMs) to detect any differences among treatments (high-*p*CO_2_, ambient). Subsequently, we analysed total abundances and abundances from the most frequent mesozooplankton species using general additive mixed models (GAMMs) to analyse the effect of the treatments as well as temporal trends. We compared the development of the community between treatments by a non-metric multidimensional analysis (NMDS) followed by a similarity analysis (ANOSIM). Finally, focusing on the most abundant species in the mesocosms (*P*. *acuspes*), we compared productivity and females’ condition between treatments by using GLMs.

Mesozooplankton diversity in mesocosms was calculated by using the Simpson’s Diversity Index (*D*) for finite communities. This index ranges from 0 to 1, and it is adapted to the form 1-*D* for a more intuitive interpretation of the results, thus higher values indicate higher sample diversity. Males, females and copepodites of the same copepod species were pooled together. Nauplii were assumed to be *P*. *acuspes* since this species accounted for > 90% of the copepod abundance during the whole experiment. General linear models (GLMs) were fitted to the Simpson’s indices to determine the dependence of diversity 1-*D* on time and *p*CO_2_. Calculations of *D* were performed in the vegan package of the R environment [60].

A multivariate analysis (NMDS) was used to describe the changes in the mesozooplankton community throughout the mesocosm experiment. NMDS is an ordination technique which represents, in an *n*-dimensional space, the dissimilarities obtained from an abundance data matrix [61]. NMDS takes a rank based approach, being more robust to datasets like the one used here, but as a consequence all the information about the magnitude of distances is lost. NMDS is therefore useful to represent the dissimilarities, and assess the influence of the treatment in the evolution of the community. However, due to the lack of magnitude, this technique is not ideal to evaluate the influence of environmental gradients on community changes [62]. The treatment effect was assessed by using permutation tests on the community position in the NMDS space, by checking if the area of clusters formed by the treatment in the NMDS were smaller than randomized samples of the same size [62]. In a complementary approach, we applied an ANalysis Of SIMilarity (ANOSIM) test [63] as a post-analysis to compare the mean of ranked dissimilarities between treatments (high-*p*CO_2_, ambient) to the mean of ranked dissimilarities within treatments. This analysis tests the assumption of ranges of (ranked) dissimilarities within groups are equal, or at least very similar [64].

Only those species that were present in at least one of the mesocosms for more than nine sampling days (2/3 of the number of days sampled) were used for temporal trends and multivariate analyses. By this criterion, the species selected for the analyses were: the hydromedusae *Aglantha digitale* and *Hybocodon prolifer*, and the females, males and copepodites of the copepod species *Oithona similis*, *Temora longicornis*, and *P*. *acuspes*. The aggregated copepod nauplii (pooled in one group and not identified to species level) were also included in these analyses.

To describe the temporal trends of each species during the mesocosm experiment we used GAMMs [61, 65] with a Poisson distribution and with a logarithmic transformation. Four different kinds of models were fitted to each abundance group (Table 1). Each of these models allowed the temporal trends to vary differently between treatments, representing (a) no difference between treatments (*α + f*), (b) differences in temporal trends but not in abundance (*α + f*_*T*_) (c) difference in absolute abundance but not in temporal trends (*α*_*T*_ *+ f*) and (d) difference both in absolute abundance and temporal trends (*α*_*T*_ *+ f*_*T*_). In this way potential differences between *p*CO_2_ and ambient mesocosms could be detected as either increase/decrease of overall abundance or changes in phenology. All models were fitted with an autocorrelation structure of first order to account for temporal autocorrelation in the data, and the specific mesocosm was used as a random intercept as the focus of the analyses was not the differences between mesocosms, but between treatments [61]. The models were compared by means of the Akaike Information Criterion (AIC). AIC takes into account both the goodness of fit of the model and model complexity, with lower AIC values indicating models with a better ratio between the explained variance and the number of variables [65]. For each species, the model with the lowest AIC was considered to better represent the temporal trends during the experiment, while avoiding overfitting the data.

**Table 1:** Generalized Additive Mixed Model (GAMM) structures.

*α + f*	Temporal trend and absolute abundances are treatment-independent *(*Model *Trtmt_indep)*
*α + f*_*T*_	Temporal trends depend on the treatment, but absolute abundances are treatment independent *(*Model *Trtmt_trend)*
*α*_*T*_ *+ f*	Absolute abundances depend on the treatment, temporal trends are treatment independent *(*Model *Trtmt_absAb)*
*α*_*T*_ *+ f*_*T*_	Both absolute abundances and temporal trends are affected by the treatment *(*Model *Trtmt_absAb_trend)*

In the case of copepods, we analysed the effects of the end-of-century *p*CO_2_ treatment on *P*. *acuspes* productivity by estimating a *nauplii-to-adult* ratio. Afterwards, GLMs were fitted to these ratios. The differences in the physiological and reproductive condition of *P*. *acuspes* females were analysed by GLMs comparing the potential effect of treatment and month in respiration rates, carbon content, prosome length, clutch size and hatching success. The effect of the time of the year (March and May), treatment and their interaction was considered in the models.

We used R (version 3.0.2, [66]) to fit abundances data with the GAMMs and GLMs. The significance level for all statistical analysis was set to *p* < 0.05.

## 3 Results

### 3.1 Mesozooplankton community: Composition, diversity and development

The mesozooplankton community comprised 27 different species and taxonomic groups (for a complete taxon list, see Table 2). The morphological classification of the most abundant groups (copepods and hydromedusae) was consistent with the genetic analyses conducted during the experiment (see [55] for more details). Copepods were the most abundant group throughout the experiment, representing 93–97% of the total abundances. *P*. *acuspes* was the dominant species in terms of abundance; based on the sum of adults and copepodites, *P*. *acuspes* represented 99.9% of the total copepod population at the beginning of the experiment and 33.6% at the end. Together with *P*. *acuspes*, only two other copepod species (*T*. *longicornis*, *O*. *similis*) and two hydromedusae (*A*. *digitale*, *H*. *prolifer*) were regularly recorded in our quantitative analyses. Other copepods and hydromedusae, polychaetae, chaetognatha, and appendicularians, as well as echinodermata, pteropoda, fish (larvae, eggs), bivalvia, cirripedia, and cladocera were rare (counted in less than 2/3 of the number of days sampled) or very rare (recorded in less than 3 sampling days during the experiment) in the studied community.

**Table 2:** Complete list of species and taxa present in the mesocosms registered throughout the study period. Based on our records, species were classified as common (recorded on at least 9 sampling days, hence used for the GAMM analyses), rare (counted on 3 to 9 sampling days) or very rare (on less than 3 sampling days). C = common, R = rare, VR = very rare.

	Taxonomic groups	Records
1	*Aglantha digitale*	C
2	*Hybocodon prolifer*	C
3	*Sarsia tubulosa*	VR
4	*Rathkea octopunctata*	VR
5	*Obelia* sp.	VR
6	*Phialella quadrata*	VR
7	Bivalvia	VR
8	Pteropoda	R
9	Polychaeta	R
10	*Evadne* sp.	R
11	*Podon* sp.	R
12	Copepod nauplii	C
13	*Pseudocalanus acuspes*	C
14	*Temora longicornis*	C
15	*Oithona similis*	C
16	*Acartia clausi*	R
17	*Tisbe* sp.	R
18	*Centropages* cf. *hamatus*	R
19	*Calanus* sp.	VR
20	*Monstrilla* sp.	VR
21	*Ectinosoma* sp	R
22	*Parasagitta elegans*	R
23	Cirripedia	R
24	Ophiopluteus larvae	VR
25	Sea urchin larvae and juveniles	R
26	*Oikopleura dioica*	R
27	Teleostei (fish larvae)	VR

**Fig 2:**
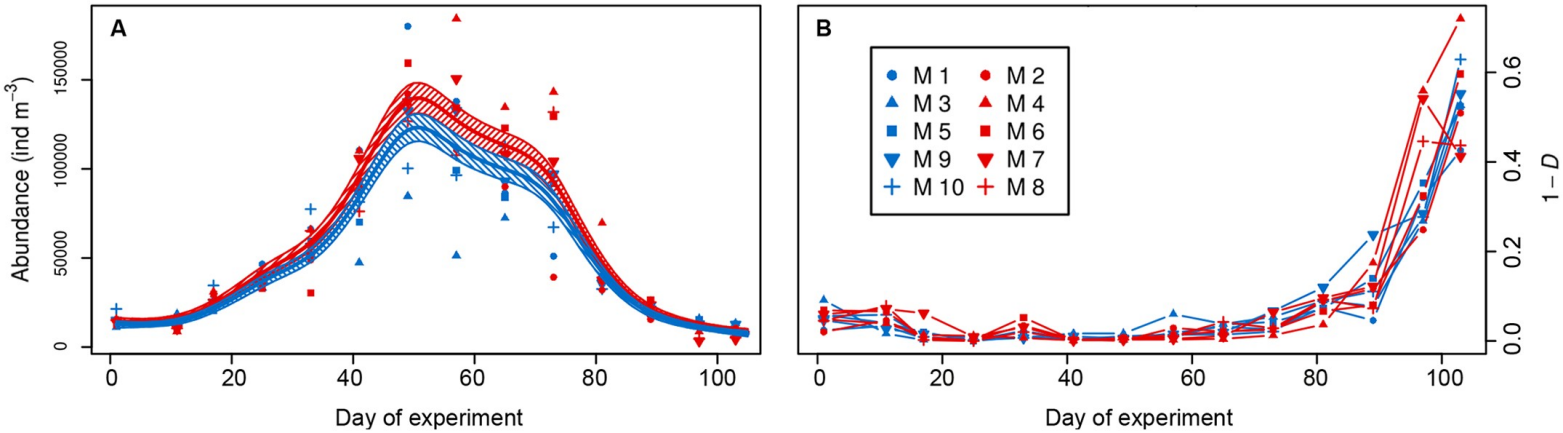
Mesozooplankton community. A) Mesozooplankton abundances. Solid lines = prediction from Generalized Additive Mixed Models (GAMMs) (smoother trends *p*-value < 0.05) with ambient and high-*p*CO_2_ mesocosms separately; striped area = confidence interval. B) Simpson’s Diversity Index (1-*D*) in relation to *p*CO_2_ levels within the mesocosms along the study period. Symbols and colours (blue = ambient; red = high-*p*CO_2_ treatment) identify each mesocosm.

Mesozooplankton abundances (Fig 2A) increased after the first phytoplankton built-up (t_17_), and decreased during the phytoplankton post-bloom phase (t_41_-t_77_) and before microzooplankton increase (t_81_) (Fig 1C and 1D). GAMM analysis showed a treatment effect in total mesozooplankton abundances, which were higher under acidification scenarios (*Trtmt_abdAb*, Table 3). Averaged total catch (M1-M10) at the beginning of the experiment (t_1_) was 14571 ± 2857 individuals per m^3^, reached maximum in t_49_ (136342 ± 24451 individuals per m^3^), to decrease until minimum levels at t_103_ (9497 ± 3111 individuals per m^3^). Mesozooplankton biodiversity (1-*D*) was low during the experiment (Fig 2B), with average values of 0.094 ± 0.018 in ambient conditions and 0.098 ± 0.043 in the high-*p*CO_2_ mesocosms. No differences between ambient conditions and high-*p*CO_2_ treatment were observed (non-significant effect of treatment in a GLM). Independently from the *p*CO_2_ treatment, Simpson’s index (1-*D*) stayed below 0.1 in both treatments until t_81_. Then the index increased, with maxima on t_103_ (0.552 ± 0.045 in ambient and 0.535 ± 0.126 in high-*p*CO_2_, respectively).

**Table 3:** Mesozooplankton community models selection. Generalized Additive Mixed Models (GAMMs) for the mesozooplankton community: a) *α + f*, no difference between treatments (Model *Trtmt_indep*), b) *α + f*_*T*_, *p*CO_2_ treatment effect on temporal trends but not in abundance (Model *Trtmt_trend*), c) *α*_*T*_ *+ f*, *p*CO_2_ treatment effect on absolute abundance but not on temporal trends (Model *Trtmt_absAb*) and d) *α*_*T*_ *+ f*_*T*_, treatment causes differences both in absolute abundance and seasonal trends (Model *Trtmt_absAb_trend*). Only those species that were present in at least one of the mesocosms more than 9 days (2/3 of the number of days sampled) and only convergent models were used for this analyses. The smoother of all selected models had a *p*-value < 0.05. For each species, the model with the lowest AIC (boldface) was considered to better represent the temporal trend during the experiment. Hyphens (-) indicate non-convergent models.

Taxa	Model type	R^2^	AIC	Taxa	Model type	R^2^	AIC
nauplii	***Trtmt_indep***	**0.855**	**257.797**	*T*. *longicornis* copepodites	*Trtmt_indep*	0.123	544.681
	*Trtmt_trend*	0.855	278.645		*Trtmt_trend*	0.127	540.113
	*Trtmt_absAb*	0.859	258.568		*Trtmt_absAb*	0.169	544.147
	*Trtmt_absAb_trend*	0.854	279.925		***Trtmt_absAb_trend***	**0.122**	**536.422**
*P*. *acuspes* ♀	***Trtmt_indep***	**0.441**	**189.89**	*O*. *similis* ♀	*Trtmt_indep*	0.558	463.501
	*Trtmt_trend*	0.491	195.135		***Trtmt_trend***	**0.583**	**445.861**
	*Trtmt_absAb*	0.443	191.887		*Trtmt_absAb*	0.552	465.903
	*Trtmt_absAb_trend*	0.5	197.739		*Trtmt_absAb_trend*	0.582	448.497
*P*. *acuspes* ♂	***Trtmt_indep***	**0.564**	**282.254**	*O*. *similis* ♂	*Trtmt_indep*	0.605	484.982
	*Trtmt_trend*	0.586	307.326		*Trtmt_trend*	0.635	482.307
	*Trtmt_absAb*	0.573	283.754		*Trtmt_absAb*	0.599	482.24
	*Trtmt_absAb_trend*	0.586	310.298		***Trtmt_absAb_trend***	**0.633**	**479.176**
*P*. *acuspes* copepodites	*Trtmt_indep*	0.727	210.277	*O*. *similis* copepodites	***Trtmt_indep***	**0.767**	**447.67**
	*Trtmt_trend*	0.752	232.495		*Trtmt_trend*	0.759	469.749
	***Trtmt_absAb***	**0.76**	**209.844**		*Trtmt_absAb*	0.766	449.509
	*Trtmt_absAb_trend*	0.75	234.226		*Trtmt_absAb_trend*	0.758	471.615
*T*. *longicornis* ♀	*Trtmt_indep*	-	-	*A*. *digitale*	*Trtmt_indep*	0.118	735.989
	*Trtmt_trend*	-	-		***Trtmt_trend***	**0.114**	**734.663**
	***Trtmt_absAb***	**0.044**	**635.237**		*Trtmt_absAb*	0.11	736.248
	*Trtmt_absAb_trend*	0.197	668.866		*Trtmt_absAb_trend*	0.11	739.801
*T*. *longicornis* ♂	***Trtmt_indep***	**0.157**	**614.175**	*H*. *prolifer*	*Trtmt_indep*	0.083	811.073
	*Trtmt_trend*	-	-		*Trtmt_trend*	0.151	764.543
	*Trtmt_absAb*	0.148	615.588		*Trtmt_absAb*	0.19	812.093
	*Trtmt_absAb_trend*	0.069	614.303		***Trtmt_absAb_trend***	**0.173**	**764.455**
Total catch	*Trtmt_indep*	0.852	92.57				
	*Trtmt_trend*	0.867	104.36				
	***Trtmt_absAb***	**0.868**	**91.95**				
	*Trtmt_absAb_trend*	0.866	106.35				

**Fig 3:**
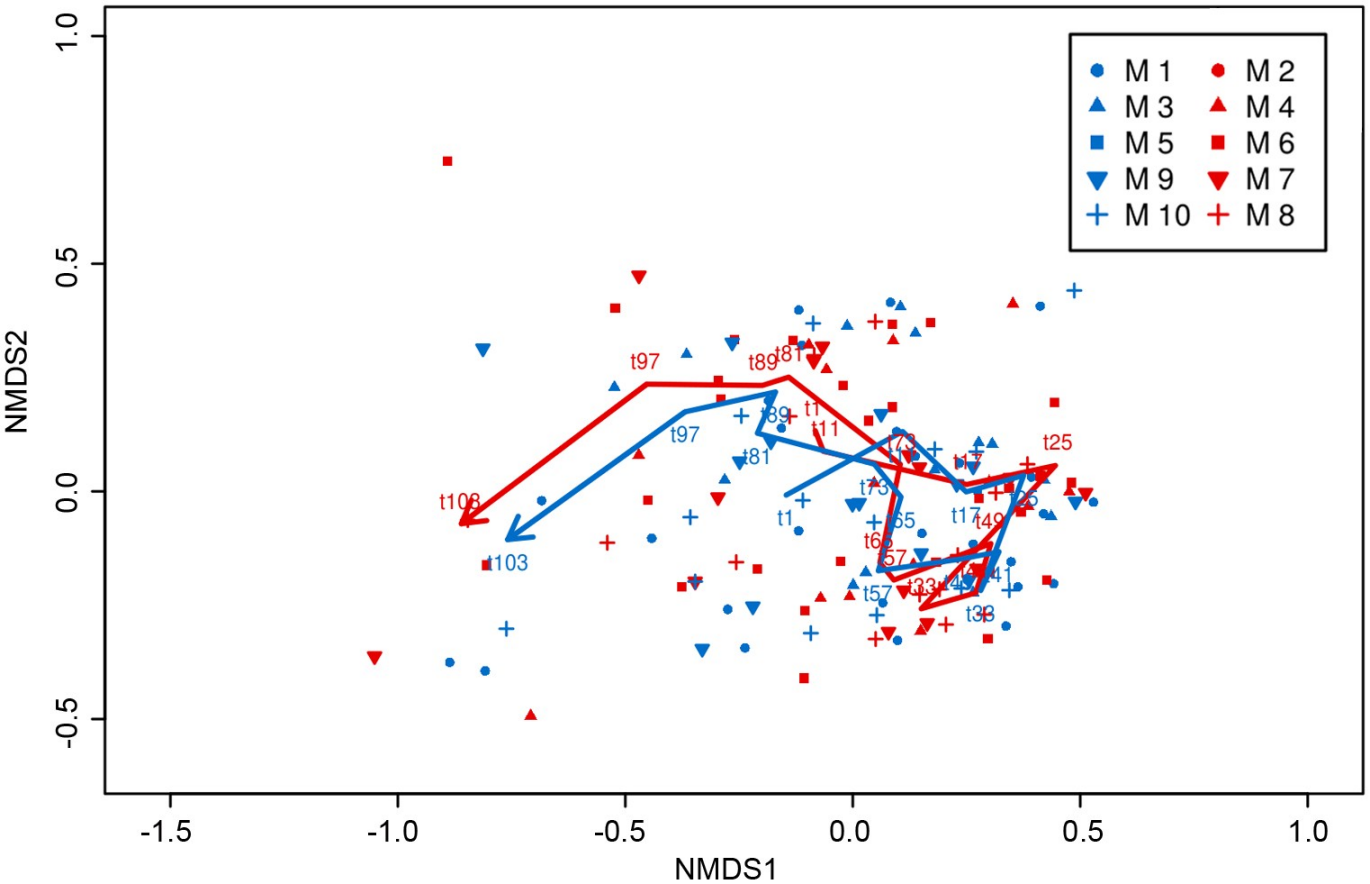
Non-metric Multidimensional Scaling analysis (NMDS) of the mesozooplankton community (stress value = 0.17). Colour code: red = treatment (~760 μatm *p*CO_2_), blue = control (ambient conditions). Sampling days represented as *t-day*; lines represent patterns. The underlying data implemented in the analysis are shown in Fig 1.

The 2-dimensional representation of the community did not show different patterns between treatments (Fig 3). Permutation tests (with 999 permutations) did not show the areas (i. e. clusters of samples) representing the treatment to be significantly smaller than randomized areas, indicating no treatment effect in the ordination. On the contrary, areas representing the sampling day (Fig 3) were significantly smaller than randomized areas using the same test. This result indicates clear community differences throughout the study period. Results from the ANOSIM test (*p*-value = 0.322) matched with the NMDS, suggesting that there was no significant difference between the community development under the high-*p*CO_2_ treatment and the ambient conditions.

### 3.2 Species abundances

**Fig 4:**
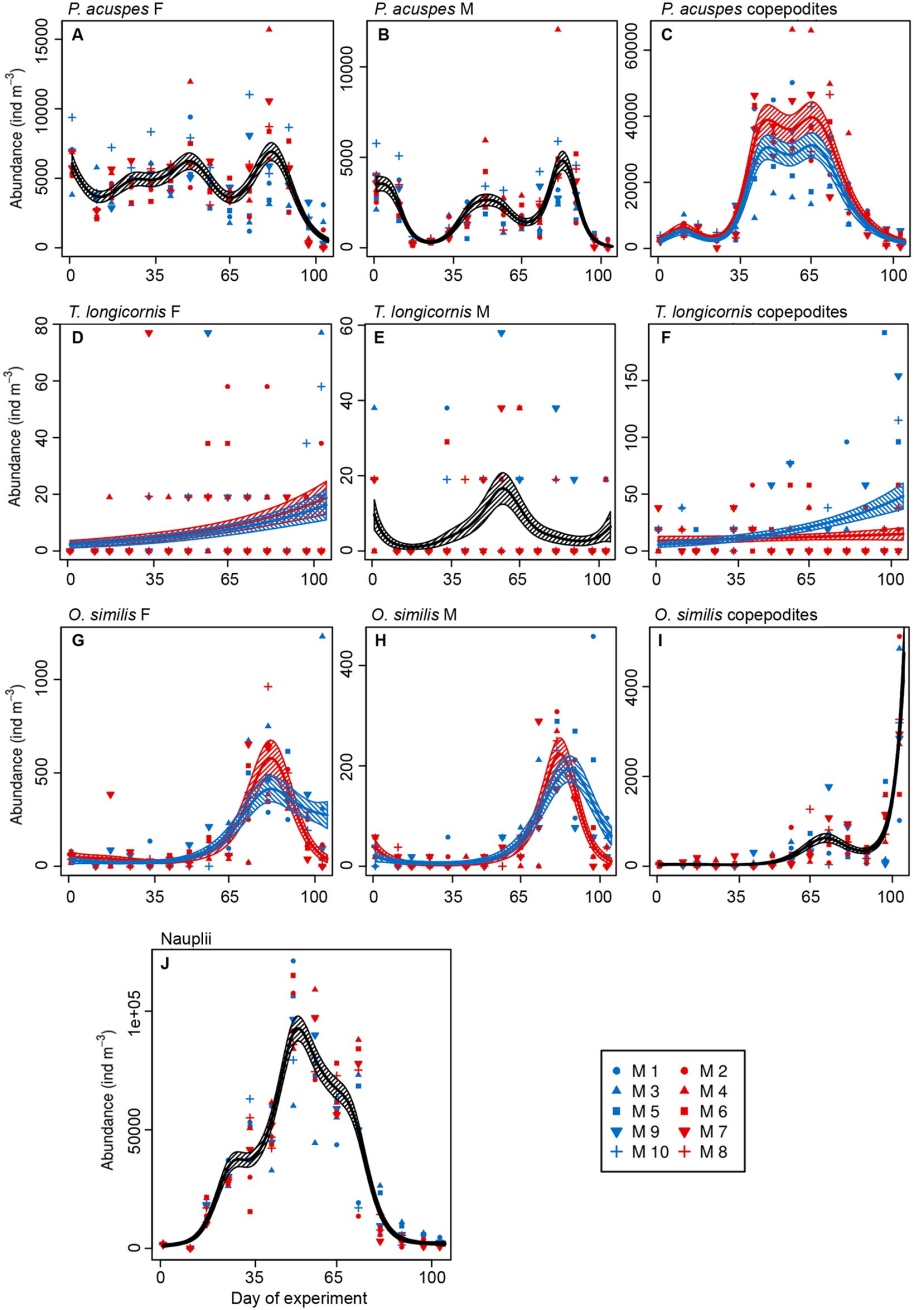
Copepod abundances along the study period. A) *P*. *acuspes* females, B) *P*. *acuspes* males, C) *P*. *acuspes* copepodites, D) *T*. *longicornis* females, E) *T*. *longicornis* males, F) *T*. *longicornis* copepodites, G) *O*. *similis* females, H) *O*. *similis* males, I) *O*. *similis* copepodites, J) nauplii. Colour code: red = treatment (~760 μatm *p*CO_2_), blue = control (ambient conditions). M = mesocosms. Solid lines = prediction from Generalized Additive Mixed Models (GAMMs) (smoother trends *p*-value < 0.05) with the ambient and high-*p*CO_2_ mesocosms shown separately; striped area = confidence interval. Black lines indicate that the prediction of the model for high-*p*CO_2_ treatment and ambient conditions are the same.

**Fig 5:**
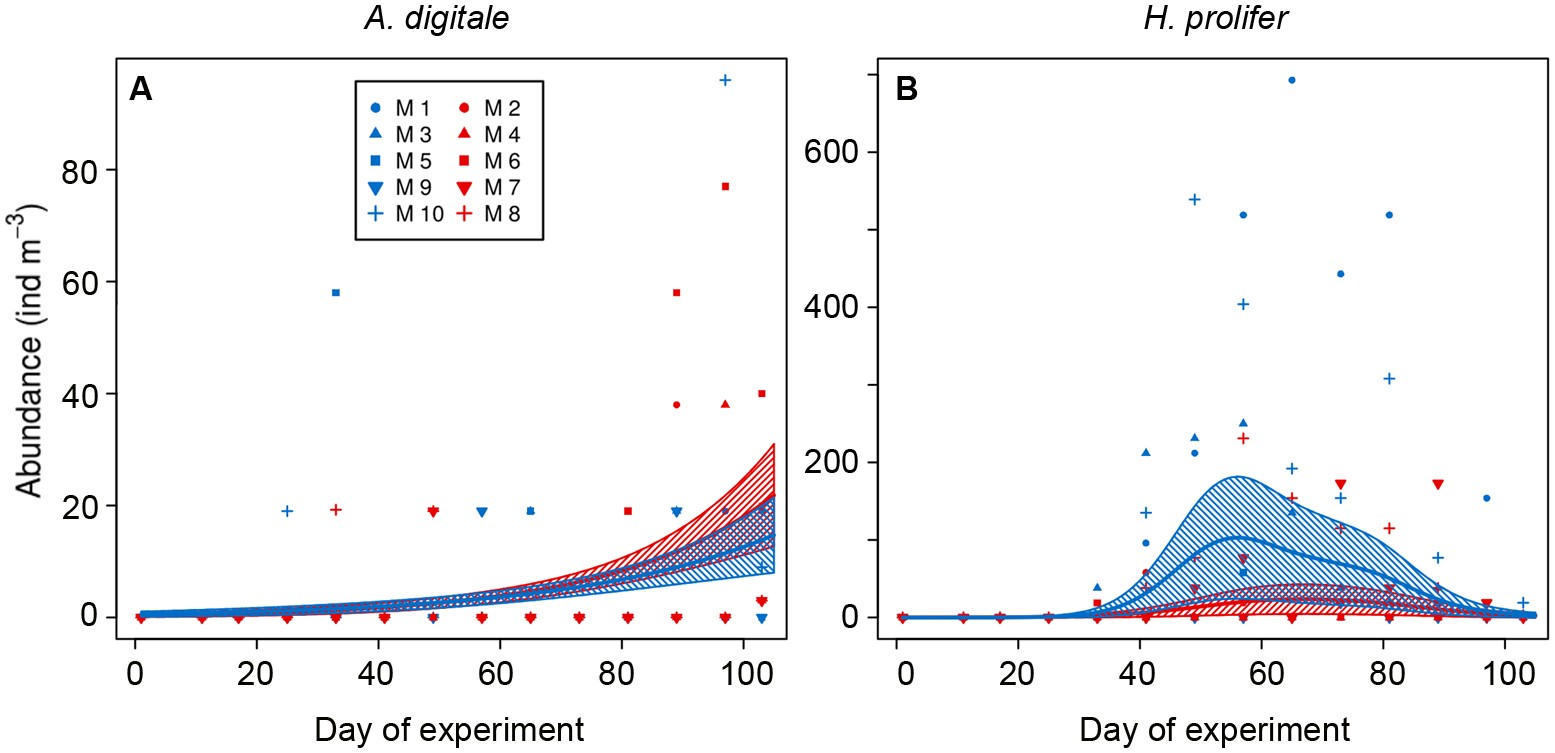
Hydromedusae abundances along the study period. A) *A*. *digitale*, B) *H*. *prolifer*. Colour code: red = treatment (~760 μatm *p*CO_2_), blue = control (ambient conditions). M = mesocosms. Solid lines = prediction from Generalized Additive Mixed Models (GAMMs) (smoother trends *p*-value < 0.05), with the ambient and high-*p*CO_2_ mesocosms shown separately; striped area = confidence interval.

Temporal trends of the selected species were analysed by using GAMMs (Figs 4 and 5; Table 3). The model selection procedure discerned whether there was a difference in the temporal trends and abundances in between the two different treatments (i.e. high or ambient *p*CO_2_).

There was no *p*CO_2_ effect on the abundance of adult *P*. *acuspes* and *T*. *longicornis* but copepodite stages of both species responded to increased *p*CO_2_. *P*. *acuspes* adults did not show differences in abundances nor in temporal trends between treatments (Table 3 *Trtmt_indep* for both males and females; Fig 4A and 4B). However, the absolute abundance of *P*. *acuspes* copepodites differed between treatments, being higher under the high-*p*CO_2_ treatment (Table 3 *Trtmt_absAb*; Fig 4C). Abundance of *T*. *longicornis* adults did not show a difference between treatments (Fig 4D and 4E); even though the selected model showed slightly higher abundances of *T*. *longicornis* females in the high-*p*CO_2_ mesocosms (Table 3 *Trtmt_absAb*; Fig 4D), the confidence intervals of the modelled abundances were overlapping throughout the study period. This indicates that the difference were small, and probably caused by extreme values at the end of the experiment. Only *T*. *longicornis* copepodites (Table 3 *Trtmt_absAb_trend*; Fig 4F) showed different absolute abundances and a different temporal trend between treatments, being more abundant in the ambient *p*CO_2_ mesocosms, particularly during the last 20 days of the study. *O*. *similis* adults negatively responded to the elevated *p*CO_2_ conditions with an earlier abundance decrease towards the end of the experiment (Fig 4G and 4H). In case of *O*. *similis* males the absolute abundance and the temporal trend were negatively affected by the high-*p*CO_2_ treatment (Table 3 *Trtmt_absAb_trend*). However, this effect was not detected on *O*. *similis* copepodites (Table 3 *Trtmt_indep*; Fig 4I), which showed no significant difference between both treatments. Copepod nauplii, the most abundant group in the mesozooplankton (Fig 4J), did not show a difference in temporal trends nor abundance between treatments (Table 3 *Trtmt_indep*). When analysing abundances in certain time-points, we could detect different *p*CO_2_ effects that were not detected by the GAMMs. In the case of *P*. *acuspes*, adult copepods were significantly more abundant on t_81_ (*t*-test, *p*-value = 0.010), but the effect disappeared afterwards. Different responses were also observed on nauplii abundances, which were significantly higher under high-*p*CO_2_ conditions between t_49_ and t_65_ (*t*-test, *p*-value = 0.03), whilst we did not detect differences in abundances between treatments when analysing abundances from t_65_ until the end of the experiment (*t*-test, *p*-value = 0.622).

In the case of both hydromedusa species, we also detected species-specific *p*CO_2_ effects (Fig 5, Table 3). Under the high-*p*CO_2_ treatment, *H*. *prolifer* abundance was lower; the GAMM detected an effect not only on the temporal trend, but also on the abundances of this species (Table 3 *Trtmt_absAb_trend*). The model representing *A*. *digitale* also showed a different temporal trend between treatments (Table 3 *Trtmt_trend*) despite of the confidence intervals overlapping of both patterns.

To sum up, after analysing the abundance of each species under high-*p*CO_2_ conditions during the whole study period we observed positive (*P*. *acuspes* copepodites, *A*. *digitale*), negative (*T*. *longicornis* copepodites, *H*. *prolifer*, *O*. *similis* adults) and no effects of elevated *p*CO_2_ (nauplii, *P*. *acuspes* and *T*. *longicornis* adults, *O*. *similis* copepodites). It is worth mentioning that the predictive power (R^2^) of these models was low in some cases (see Table 3) due to the complete absence of some species in some mesocosms. However, the models represented well the overall trend differences between treatments (Figs 4 and 5). Differences between treatments were at times significant for specific time periods.

### 3.3 *P*. *acuspes*: Productivity and females’ condition

**Fig 6:**
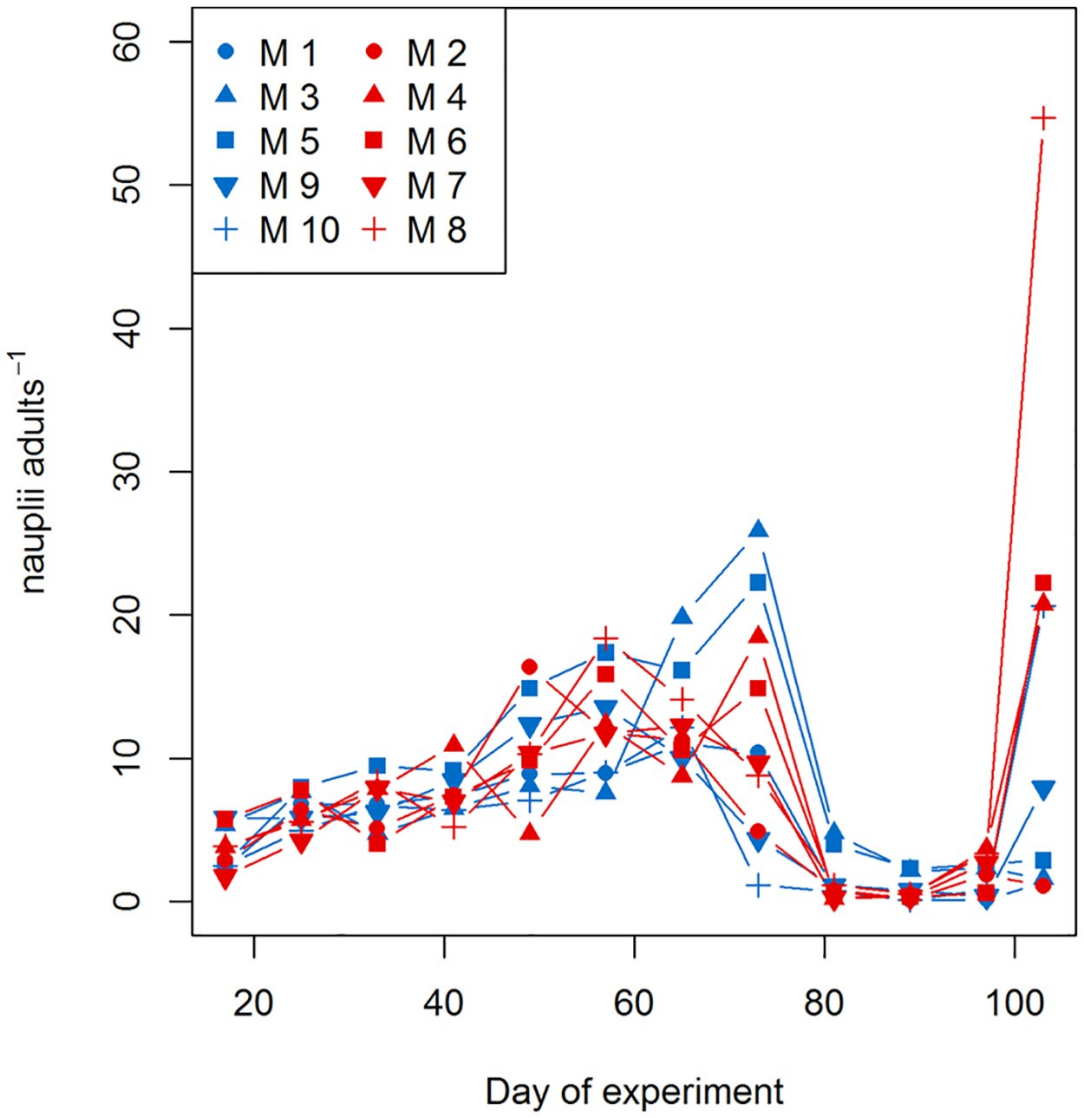
*P*. *acuspes* productivity in relation to *p*CO_2_ levels along the study period. Symbols and colours (blue = ambient; red = high-*p*CO_2_ treatment) identify each mesocosm. Production estimated as the ratio between nauplii and adults. *P*. *acuspes* nauplii abundances were estimated from the relative abundances of *P*. *acuspes* in relation to total copepod abundances per sampling day and mesocosm.

Copepod productivity was assessed by computing the ratio between nauplii and adults for the most abundant species, *P*. *acuspes*. We calculated the *nauplii-to-adult* ratio from t_17_ until the end of the experiment, since the fraction < 200 μm was preserved only from t_17_ on. At a significance level of 0.05, no differences in this ratio between the ambient and high-*p*CO_2_ treatment (GLM, *p*-value = 0.576), but a significant effect of time (GLM, *p*-value < 0.001) was detected. Productivity increased from the beginning of the experiment until t_65_ or t_73_ independently of the *p*CO_2_ treatment (see Fig 6), and rapidly decreased afterwards. A second increase in the productivity was detected from t_97_, with the highest ratios in some of the high-*p*CO_2_ mesocosms.

**Fig 7:**
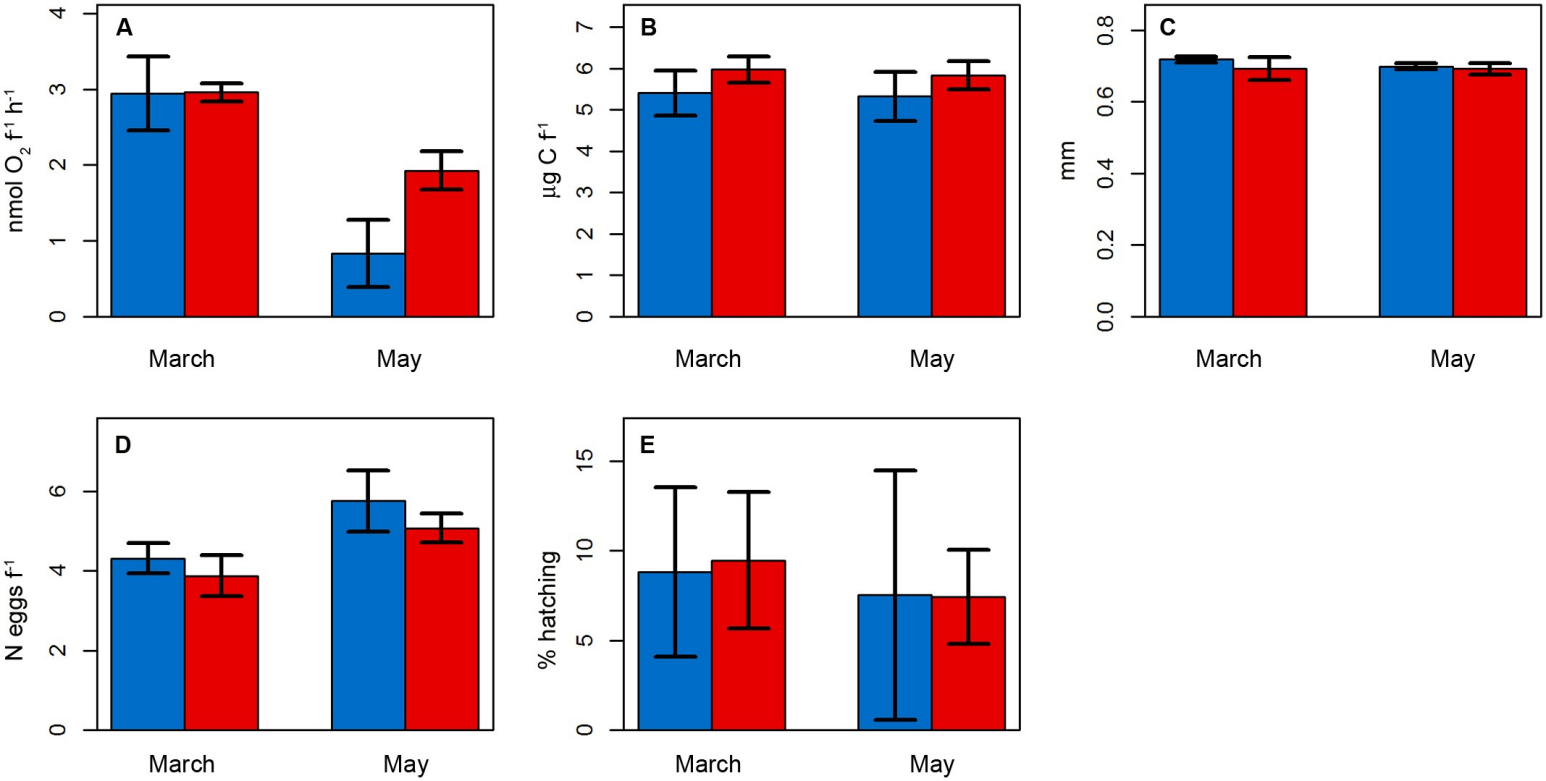
*P*. *acuspes* females’ condition. General Linear Models (GLMs) comparing the potential *p*CO_2_ effect on *P*. *acuspes* females: A) respiration rate, B) carbon content, C) prosome length, D) clutch size at the beginning of the incubation (0h), E) hatching success after 48h incubation. Error bars represent standard deviation. Colour code: red = treatment (~760 μatm *p*CO_2_), blue = control (ambient conditions). March = t_19_ (first phytoplankton bloom), May = t_59_ (decline phase of the second phytoplankton bloom).

Regarding the *P*. *acuspes* females’ condition, none of the physiological and reproductive parameters investigated (respiration, carbon content, prosome length, clutch size, hatching success) showed a significant difference between treatments, nor in the interaction between month and treatment (*p*-value > 0.05; Fig 7, Table 4). However, significant differences between the first (March, t_19_: first phytoplankton bloom) and the second experiment (May, t_59_: second phytoplankton bloom) were observed. Respiration rate (Fig 7A) was lower during May compared to March (*p*-value = 0.001). Females’ carbon content and prosome length, as well as the hatching success after 48h incubation (Fig 7B, 7C and 7E) were not different between months, nor between *p*CO_2_ conditions. Yet, at the beginning of the incubations (0h), clutch size (Fig 7D) was significantly higher in May (*p*-value = 0.021). None of the interactions between *p*CO_2_ treatment and month rendered in a significant effect on the studied variables.

**Table 4:** Results from *P*. *acuspes* females’ condition experiment. Generalized Linear Models (GLMs) based on two laboratory experiments (March, May), *n* = 120 females per experiment. Boldface represent *p*-values < 0.05.

Respiration	Estimate	Std.Error	t-value	p-value
(Intercept)	5.035	0.786	6.406	0
*p*CO_2_ treatment	0.553	0.37	1.492	0.154
month	-0.786	0.185	-4.246	**0.001**
**Carbon content**				
(Intercept)	5.586	0.958	5.829	0
*p*CO_2_ treatment	0.541	0.452	1.198	0.247
month	-0.056	0.226	-0.246	0.808
**Prosome length**				
(Intercept)	0.728	0.039	18.875	0
*p*CO_2_ treatment	-0.016	0.018	-0.895	0.383
month	-0.005	0.009	-0.536	0.599
**Clutch size (0h)**				
(Intercept)	2.394	1.103	2.17	0.044
*p*CO_2_ treatment	-0.563	0.52	-1.082	0.294
month	0.661	0.26	2.542	**0.021**
**Hatching success**				
(Intercept)	11.465	9.875	1.161	0.262
*p*CO_2_ treatment	0.275	4.655	0.059	0.954
month	-0.823	2.328	-0.354	0.728

## 4 Discussion

During this winter-to-summer experiment on the effect of ocean acidification on plankton communities, we did not detect an effect of *p*CO_2_ on either the diversity of the mesozooplankton community, nor on its development as a whole. At first sight, this may seem surprising as some taxa showed a response to OA, where others did not. The most parsimonious explanation for this apparent contradiction is the strong dominance of the copepod *P*. *acuspes*. As a result, changes in the relative composition of the community were small and were not be picked up by relatively coarse indicators such as Simpson’s Diversity or rank-based methods such as NMDS. Only on the last two sampling days, when *P*. *acuspes* abundances declined strongly, a trend towards a higher diversity under high-*p*CO_2_ conditions became visible (Figs 2B and 3), and the communities under the two treatments diverged (observed also for microzooplankton [48]). Potentially this indicates a long-term effect of high *p*CO_2_ on the communities, but this is impossible to say as, at that time the mesocosm set-up started to deteriorate and the experiment was terminated.

Unlike previous mesocosms studies focusing on the effect of OA on natural coastal plankton communities in the Arctic [67] and the Baltic [39], we detected a positive *p*CO_2_ effect on the total mesozooplankton abundance from Gullmar Fjord. This effect was mostly caused by the CO_2_-driven increase in the abundances of *P*. *acuspes* copepodites. This was somewhat unexpected, as previously work on the same species from the same location [42, 68] found significant negative *p*CO_2_ effects on egg production and metabolism. The two studies cited above were highly controlled laboratory experiments, where the copepods were cultured under uniform environmental conditions (except for the *p*CO_2_ treatments) and offered identical prey in all treatments. Thus, the effects observed were directly caused by changes in carbonate chemistry of the water as all other environmental factors were identical. In semi-natural experiments such as the one described here, these effects are easily masked, either through bottom-up effects (changes in the availability or quality of the food), or as a result of top-down effects (changes in predation rates). In our two condition experiments we excluded the latter effects, and focused on the effects of the overall growing conditions in the mesocosms. In contrast to the laboratory experiments cited above, we did not find significant differences in the physiological condition of *P*. *acuspes* females between ambient and high-*p*CO_2_ treatments (Fig 7). Secondary production in *P*. *acuspes* followed a temporal trend, with higher clutch sizes and nauplii abundances on t_59_ (May), responding to higher phytoplankton concentration (chl*a*) and microzooplankton biomass. However, this increase in food quantity might not have been coupled with food quality to maintain the copepod population in the mesocosms, which increased from ~260 ± 5 copepods L^-1^ (t_19_) to ~1245 ± 32 copepods L^-1^ (t_59_). This could explain lower respiration rates in May than in March [69, 70]. Potential food items for copepods on t_19_ (March) consisted mainly of phytoplankton between 5 and 40 μm and microzooplankton biomass below 2 μg C L^-1^ before the first phytoplankton bloom in the mesocosms [48, 71]. On t_59_ the entire mesocosms system was dominated by *Coscinodiscus concinnus* (representing 47% of the biomass) and the nanophytoplankton fraction (accounting for 21%) [71], both largely outside the food spectrum of *P*. *acuspes*. Microzooplankton biomass was ~12 μg C L^-1^ on t_59_ [48], but might not have been enough to supply the whole *P*. *acuspes* population, so copepods might have searched for alternative food sources such as sinking material. In fact, the decrease in adults from t_97_ in all mesocosms matched high resolution images taken from sediment trap material, where high abundances of adult *P*. *acuspes* were found (Tim Boxhammer, pers. comm.). This observation suggest that, towards the end of the experiment, copepods might have migrated downward searching for food and stayed close to the sediment traps, as previously observed in a mesocosms experiment in a Norwegian fjord [72].

In view of the result of the two laboratory experiments, where we observed no effects of *p*CO_2_ on egg production, the most plausible explanation for the higher *P*. *acuspes* abundances under the high-*p*CO_2_ treatment is a community CO_2_-driven bottom-up effect [10, 12, 73]. This is not a contradiction, as in the laboratory experiments we specifically looked at the memory *p*CO_2_ effect on the clutch, which was not expected to be affected by the 48h food deprivation regime [74]. Thus, the higher abundance of *P*. *acuspes* copepodites was probably fuelled by phytoplankton community responses to high-*p*CO_2_ conditions during our mesocosms experiment. Higher primary production [75] and higher chl*a* levels under high-*p*CO_2_ [45] resulted in higher copepodite abundances. Interestingly, this CO_2_-driven increase in copepodite abundances did not result in higher abundances of adults later in the season except on t_81_, when adult *P*. *acuspes* were significantly more abundant under high-*p*CO_2_ conditions. The most plausible explanation for this trend in adult *P*. *acuspes* abundance after t_81_ is, apart from the potential downward migration as indicated above, that the level of top-down control through herring larvae was different, with higher predation pressure in high-*p*CO_2_ mesocosms. As detailed in Sswat et al. [57], after hatching on ~t_63_, herring larvae would have gradually switched from endogenous to exogenous feeding, preying then firstly on nauplii and ciliates, afterwards increasing the size of their prey gradually with their own body size until they reached copepodites (~t_65_-t_81_) and finally adults (~t_81_-t_105_)[76–78]. From t_77_ (14^th^ day post-hatching, DPH) survival of herring larvae was significantly higher in the high-*p*CO_2_ mesocosms [57], which would imply higher grazing pressures on *P*. *acuspes*. Since consumption rates of smaller larvae are much lower than those of larger ones, we would have only detected a top-down effect of the herring larvae on adult abundance at the end of the experiment. This, together with a more intensive feeding activity by herring larvae because of the higher larvae survival rates under the acidic treatment [57], could have caused lower abundances of adult *P*. *acuspes* relative to the opposite pattern in the copepodites.

In the case of *T*. *longicornis*, no effects of *p*CO_2_ were observed on the adults but copepodites were more abundant under ambient conditions, especially during the last 20 days of the experiment (Table 3, Fig 4D–4F). This finding fits to the last two sampling days divergence between treatments in the NMDS analysis (Fig 3), which points to a different development of the community under ambient and high-*p*CO_2_ conditions. The particular tolerance in *T*. *longicornis* female reproductive fitness to end-of-century *p*CO_2_ scenarios had already been described by McConville et al. [27]. However, the higher abundances of *T*. *longicornis* copepodites observed in ambient conditions suggest that this tolerance might be diminished in early life stages, as previously observed in other calanoid copepods [29, 79].

Our results suggest a negative effect of *p*CO_2_ on adult *O*. *similis*, which were more abundant under ambient conditions when considering the whole experimental period. The explanation for *O*. *similis*’ sensitivity to OA observed in adults might be in the life history of this copepod. According to Lewis et al. [33] there is a correlation between sensitivity to OA and vertical migration behaviour. Species that do not exhibit diel vertical migration behaviour (as *O*. *similis*) are typically less exposed to variation in *p*CO_2_ levels compared to other copepods and more prone to be sensitive to OA [33, 80]. For *O*. *similis*, these researchers detected reduced adult and naupliar survival under 700 and 1000 μatm *p*CO_2_. Our study would support this observation by lower *O*. *similis* adult abundances under high-*p*CO_2_ conditions. Towards the end of the experiment, however, we observed an increase in *O*. *similis* abundance, likely reacting to the increase in ciliates and dinoflagellates biomass [48]. Adults showed a significant reaction to OA with firstly higher and subsequently lower abundances in the high-*p*CO_2_ treatment. As also observed on adult *P*. *acuspes*, the differential decrease in adult *O*. *similis* within treatments from t_81_ might respond to herring larvae abundance and the size-dependent feeding activity [57, 77]. Thus considering that during the last two sampling days adults would probably be in the preferred size range for the herring larvae, the release in preying pressure on copepodites and the built-up of protozooplankton [48] might explain the final increase in copepodite abundance in both treatments.

Whilst the connection between jellyfish blooms (scyphomedusae, hydromedusae, siphonophores and ctenophores) and anthropogenic climate change remains unclear (e. g. [81, 82]), the effects of changing seawater carbonate chemistry on planktonic gelatinous species have been rarely tested. However, all results on different gelatinous zooplankton groups (schyphomedusa ephyrae [19, 83, 84], coelenterate records [85]) point to the tolerance of jellyfish to future changes in *p*CO_2_. In this study we showed for the first time the species-specific sensitivity of hydromedusae to OA. Thus *H*. *prolifer* (Anthomedusa) reacted negatively to high *p*CO_2_ by lower abundances, while *A*. *digitale* (Trachymedusa) was more abundant in the high-*p*CO_2_ treatment (Table 3, Fig 5). This result was unexpected, given the fact that *A*. *digitale* has statoliths, which could be a target for lower pH (as Richardson and Gibbons [85] also noted). Our findings suggest that hydromedusae with statoliths are not necessarily more sensitive than those without these calcium-based structures, and consequently hydromedusa statoliths might not be sensitive to OA, at least in realistic end-of-century scenarios. Further ecophysiological analyses, however, are still required for these and other hydromedusae species to confirm this hypothesis.

## Conclusion

During this study, we observed species-specific sensitivities to *p*CO_2_ in copepods and hydromedusae abundance. In the case of copepods, responses to elevated *p*CO_2_ depended also on the life-stage of the individuals, copepodites generally being the most sensitive stage. Our results point that OA could positively affect the calanoid *P*. *acuspes* by a bottom-up effect in *p*CO_2_-fuelled food webs. Nonetheless, the effect of OA on single species was not detectable in the structure or diversity of this community, probably due to the overwhelmingly dominance of *P*. *acuspes* in the studied community. Hence, under a realistic end-of-century OA scenario, the Gullmar Fjord mesozooplankton community structure is not expected to change much, although it could well be that the OA effect on copepodites would potentially affect biomass transfer to higher trophic levels in the future.

### Ethic statement

No specific permission was required for activities related to field sampling. The field location was not privately owned or protected, and neither endangered nor protected species were involved. Fish larvae experiment [57] was conducted under the ethical permission (number 332–2012 issued by the Swedish Board of Agriculture "Jordbruksverket"). Animal welfare was assured by minimization of stress from handling and treatment. Specimens were therefore anaesthetized before handling using Tricaine methanesulfonate MS-222. The CO_2_ concentrations used in this study are far below the lethal level.

## References

[1] IPCC. Climate Change 2013: The Physical Science Basis Contribution of Working Group I to the Fifth Assessment Report of the Intergovernmental Panel on Climate Change Cambridge, United Kingdom and New York, NY, USA: Cambridge University Press, 2013.

[2] Sabine CL, Feely RA, Gruber N, Key RM, Lee K, Bullister JL, et al The oceanic sink for anthropogenic CO_2_. Science. 2004;305(5682):367–71. 10.1126/science.1097403 15256665

[3] Wolf-Gladrow DA, Riebesell U, Burkhardt S, Bijma J. Direct effects of CO_2_ concentration on growth and isotopic composition of marine plankton. Tellus B. 1999;51(2):461–76.

[4] Caldeira K, Wickett ME. Oceanography: Anthropogenic carbon and ocean pH. Nature. 2003;425(6956):365- 10.1038/425365a 14508477

[5] Doney SC, Fabry VJ, Feely RA, Kleypas JA. Ocean acidification: the other CO_2_ problem. Annu Rev Mar Sci. 2009;1(1):169–92. 10.1146/annurev.marine.010908.163834 21141034

[6] Hoegh-Guldberg O, Bruno JF. The impact of climate change on the world’s marine ecosystems. Science. 2010;328(5985):1523–8. 10.1126/science.1189930 20558709

[7] Hoegh-Guldberg O, Mumby PJ, Hooten AJ, Steneck RS, Greenfield P, Gomez E, et al Coral reefs under rapid climate change and ocean acidification. Science. 2007;318(5857):1737–42. 10.1126/science.1152509 18079392

[8] Wallace RB, Baumann H, Grear JS, Aller RC, Gobler CJ. Coastal ocean acidification: The other eutrophication problem. Estuar Coast Shelf Sci. 2014;148:1–13.

[9] Gobler CJ, Baumann H. Hypoxia and acidification in ocean ecosystems: coupled dynamics and effects on marine life. Biol Lett. 2016;12(5). 10.1098/rsbl.2015.0976 PMC4892234 27146441

[10] Rossoll D, Bermudez R, Hauss H, Schulz KG, Riebesell U, Sommer U, et al Ocean acidification-induced food quality deterioration constrains trophic transfer. PLoS One. 2012;7(4). 10.1371/journal.pone.0034737 PMC3324536 22509351

[11] Boersma M, Aberle N, Hantzsche FM, Schoo KL, Wiltshire KH, Malzahn AM. Nutritional limitation travels up the food chain. Int Rev Hydrobiol. 2008;93(4–5):479–88.

[12] Cripps G, Flynn KJ, Lindeque PK. Ocean Acidification Affects the Phyto-Zoo Plankton Trophic Transfer Efficiency. PLoS One. 2016;11(4):1–15. 10.1371/journal.pone.0151739 PMC4833293 27082737

[13] Dutkiewicz S, Morris JJ, Follows MJ, Scott J, Levitan O, Dyhrman ST, et al Impact of ocean acidification on the structure of future phytoplankton communities. Nature Clim Change. 2015;5(11):1002–6.

[14] Lischka S, Büdenbender J, Boxhammer T, Riebesell U. Impact of ocean acidification and elevated temperatures on early juveniles of the polar shelled pteropod *Limacina helicina*: mortality, shell degradation, and shell growth. Biogeosciences. 2011;8(4):919–32.

[15] Orr JC, Fabry VJ, Aumont O, Bopp L, Doney SC, Feely RA, et al Anthropogenic ocean acidification over the twenty-first century and its impact on calcifying organisms. Nature. 2005;437(7059):681–6. 10.1038/nature04095 16193043

[16] Riebesell U, Zondervan I, Rost B, Tortell PD, Zeebe RE, Morel FMM. Reduced calcification of marine plankton in response to increased atmospheric CO_2_. Nature. 2000;407(6802):364–7. 10.1038/35030078 11014189

[17] Fabry VJ, Seibel BA, Feely RA, Orr JC. Impacts of ocean acidification on marine fauna and ecosystem processes. ICES J Mar Sci. 2008;65(3):414–32.

[18] Purcell JE, Uye S-i, Lo W-T. Anthropogenic causes of jellyfish blooms and their direct consequences for humans: a review. Mar Ecol Prog Ser. 2007;350:153–74.

[19] Winans AK, Purcell JE. Effects of pH on asexual reproduction and statolith formation of the scyphozoan, *Aurelia labiata*. Hydrobiologia. 2010;645(1):39–52.

[20] Turner JT. The importance of small planktonic copepods and their roles in pelagic marine food webs. Zool Stud. 2004;43(2):255–66.

[21] Landry MR, Calbet A. Microzooplankton production in the oceans. ICES Journal of Marine Science. 2004; 61:501–7.

[22] Löder MGJ, Meunier C, Wiltshire KH, Boersma M, Aberle N. The role of ciliates, heterotrophic dinoflagellates and copepods in structuring spring plankton communities at Helgoland Roads, North Sea. Mar Biol. 2011;158(7):1551–80.

[23] Calbet A, Saiz E. The ciliate-copepod link in marine ecosystems. Aquat Microb Ecol. 2005;38(2):157–67.

[24] Kleppel GS. On the diets of calanoid copepods. Marine Ecology—Progress Series. 1993;99(1–2):183–95.

[25] Boersma M, Wesche A, Hirche H-J. Predation of calanoid copepods on their own and other copepods’ offspring. Mar Biol. 2014;161(4):733–43.

[26] Kurihara H, Ishimatsu A. Effects of high CO_2_ seawater on the copepod (*Acartia tsuensis*) through all life stages and subsequent generations. Mar Pollut Bull. 2008;56(6):1086–90. 10.1016/j.marpolbul.2008.03.023 18455195

[27] McConville K, Halsband C, Fileman ES, Somerfield PJ, Findlay HS, Spicer JI. Effects of elevated CO_2_ on the reproduction of two calanoid copepods. Mar Pollut Bull. 2013;73(2):428–34. 10.1016/j.marpolbul.2013.02.010 23490345

[28] Pedersen SA, Vage VT, Olsen AJ, Hammer KM, Altin D. Effects of elevated carbon dioxide (CO_2_) concentrations on early developmental stages of the marine copepod *Calanus finmarchicus* Gunnerus (Copepoda: Calanoidae). J Toxicol Environ Health. 2014;77(9–11):535–49. 10.1080/15287394.2014.887421 24754390

[29] Meunier CL, Algueró-Muñiz M, Horn HG, Lange JAF, Boersma M. Direct and indirect effects of near-future pCO_2_ levels on zooplankton dynamics. Mar Freshw Res. 2016:-.

[30] Cripps G, Lindeque P, Flynn K. Parental exposure to elevated pCO_2_ influences the reproductive success of copepods. J Plankton Res. 2014. 10.1093/plankt/fbu052 PMC4161228 25221371

[31] Pedersen SA, Hansen BH, Altin D, Olsen AJ. Medium-term exposure of the North Atlantic copepod *Calanus finmarchicus* (Gunnerus, 1770) to CO_2_-acidified seawater: effects on survival and development. Biogeosciences. 2013;10(11):7481–91.

[32] Pedersen SA, Hakedal OJ, Salaberria I, Tagliati A, Gustavson LM, Jenssen BM, et al Multigenerational exposure to ocean acidification during food limitation reveals consequences for copepod scope for growth and vital rates. Environ Sci Technol. 2014;48(20):12275–84. 10.1021/es501581j 25225957

[33] Lewis CN, Brown KA, Edwards LA, Cooper G, Findlay HS. Sensitivity to ocean acidification parallels natural pCO_2_ gradients experienced by Arctic copepods under winter sea ice. PNAS. 2013;110(51):E4960–E7. 10.1073/pnas.1315162110 24297880 PMC3870746

[34] Isari S, Zervoudaki S, Peters J, Papantoniou G, Pelejero C, Saiz E. Lack of evidence for elevated CO_2_-induced bottom-up effects on marine copepods: a dinoflagellate–calanoid prey–predator pair. ICES J Mar Sci. 2015.

[35] Mayor DJ, Matthews C, Cook K, Zuur AF, Hay S. CO_2_-induced acidification affects hatching success in *Calanus finmarchicus*. Mar Ecol Prog Ser. 2007;350:91–7.

[36] Almén A-K, Vehmaa A, Brutemark A, Engström-Öst J. Coping with climate change? Copepods experience drastic variations in their physicochemical environment on a diurnal basis. J Exp Mar Biol Ecol. 2014;460:120–8.

[37] Dorey N, Lançon P, Thorndyke M, Dupont S. Assessing physiological tipping point of sea urchin larvae exposed to a broad range of pH. Global Change Biol. 2013;19(11):3355–67. 10.1111/gcb.12276 23744556

[38] Sala MM, Aparicio FL, Balagué V, Boras JA, Borrull E, Cardelús C, et al Contrasting effects of ocean acidification on the microbial food web under different trophic conditions. ICES J Mar Sci. 2015.

[39] Lischka S, Bach LT, Schulz KG, Riebesell U. Micro- and mesozooplankton community response to increasing CO_2_ levels in the Baltic Sea: insights from a large-scale mesocosm experiment. Biogeosciences Discuss. 2015;2015:20025–70.

[40] Rossoll D, Sommer U, Winder M. Community interactions dampen acidification effects in a coastal plankton system. Mar Ecol Prog Ser. 2013;486:37–46.

[41] Pedersen MF, Hansen PJ. Effects of high pH on a natural marine planktonic community. Mar Ecol Prog Ser. 2003;260:19–31.

[42] Thor P, Dupont S. Transgenerational effects alleviate severe fecundity loss during ocean acidification in a ubiquitous planktonic copepod. Global Change Biol. 2015;21(6):2261–71. 10.1111/gcb.12815 25430823

[43] Scheinin M, Riebesell U, Rynearson TA, Lohbeck KT, Collins S. Experimental evolution gone wild. Journal of The Royal Society Interface. 2015;12(106). 10.1098/rsif.2015.0056 PMC4424681 25833241

[44] Dupont S, Dorey N, Stumpp M, Melzner F, Thorndyke M. Long-term and trans-life-cycle effects of exposure to ocean acidification in the green sea urchin *Strongylocentrotus droebachiensis*. Mar Biol. 2012;160(8):1835–43.

[45] Bach LT, Taucher J, Boxhammer T, Ludwig A, Consortium TKK, Achterberg EP, et al Influence of ocean acidification on a natural winter-to-summer plankton succession: First insights from a long-term mesocosm study draw attention to periods of low nutrient concentrations. PLoS One. 2016;11(8):1–33. 10.1371/journal.pone.0159068 PMC4985126 27525979

[46] Riebesell U, Czerny J, von Bröckel K, Boxhammer T, Büdenbender J, Deckelnick M, et al Technical Note: A mobile sea-going mesocosm system–new opportunities for ocean change research. Biogeosciences. 2013;10(3):1835–47.

[47] Sswat M, Boxhammer T, Jutfelt F, Bach LT, Nicolai M, Riebesell U. Video of a plankton community enclosed in a “Kiel Off-Shore Mesocosm for future Ocean Simulations” (KOSMOS) during the long-term study in Gullmar Fjord (Sweden) 2013. YouTube2015.

[48] Horn HG, Sander N, Stuhr A, Algueró-Muñiz M, Bach LT, Löder MGJ, et al Low CO_2_ sensitivity of microzooplankton communities in the Gullmar Fjord, Skagerrak: evidence from a long-term mesocosm study. PLoS One. 2016;11(11):e0165800 10.1371/journal.pone.0165800 27893740 PMC5125589

[49] Razouls C, de Bovée F, Kouwenberg J, Desreumaux N. Diversity and Geographic Distribution of Marine Planktonic Copepods. 2005. p. http://copepodes.obs-banyuls.fr/en

[50] Sars GO. An Account of the Crustacea of Norway, with short descriptions and figures of all the species. Copepoda Calanoida, parts I-XIV: Bergen Museum; 1901–1903 171 p.

[51] Sars GO. An Account of the Crustacea of Norway, with short descriptions and figures of all the species. Copepoda Harpacticoida, parts I-XXXVI: Bergen Museum; 1903–1911 449 p.

[52] Sars GO. An Account of the Crustacea of Norway, with short descriptions and figures of all the species. Copepoda Cyclopoida, parts I -XIV: Bergen Museum; 1913–1918 225 p.

[53] Bouillon J, Gravili C, Pagès F, Gili J-M, Boero F. An introduction to Hydrozoa. Paris: Publications Scientifiques du Muséum; 2006.

[54] Schuchert P. The European athecate hydroids and their medusae (Hydrozoa, Cnidaria): Capitata Part 2 Rev Suisse Zool. 2010;117(3):337–555.

[55] Schuchert P. The European athecate hydroids and their medusae (Hydrozoa, Cnidaria): Filifera Part 2. Rev Suisse Zool. 2007;114(2):195–396.

[56] ICES Zooplankton Methodology Manual2000.

[57] Sswat M, Stiasny M, Taucher J, Algueró-Muñiz M, Bach LT, Jutfelt F, et al. Herring larvae can benefit from OA-induced changes in the food web. in prep. 10.1038/s41559-018-0514-6 29556079

[58] Alvarez-Fernandez S, Licandro P, van Damme CJG, Hufnagl M. Effect of zooplankton on fish larval abundance and distribution: a long-term study on North Sea herring (Clupea harengus). ICES J Mar Sci. 2015.

[59] Niehoff B, Klenke U, Hirche H-J, Irigoien X, Head R, Harris R. A high frequency time series at Weathership M, Norwegian Sea, during the 1997 spring bloom: the reproductive biology of *Calanus finmarchicus*. Mar Ecol Prog Ser. 1999;176:81–92.

[60] Oksanen J, Blanchet FG, Kindt R, Legendre P, O'Hara RG, Simpson GL, et al. R package version 2.0–4 ed2012.

[61] Zuur A, Ieno EN, Walker N, Sareliev AA, Smith GM. Mixed effects models and extensions in ecology with R. 1 ed Springer-Verlag New York 2009.

[62] Legendre P, Anderson MJ. Distance-based redundancy analysis: testing multispecies responses in multifactorial ecological experiments. Ecol Monogr. 1999;69(1):1–24.

[63] Clarke KR. Non-parametric multivariate analyses of changes in community structure. Aust J Ecol. 1993;18(1):117–43.

[64] Buttigieg PL, Ramette A. A guide to statistical analysis in microbial ecology: a community-focused, living review of multivariate data analyses. FEMS Microbiol Ecol. 2014;90(3):543–50. 10.1111/1574-6941.12437 25314312

[65] Wood SN. Generalized additive models: an introduction with R. Hall/CRC C, editor. Boca Raton, FL 2006.

[66] Team RC. R: A language and environment for statistical computing. In: Computing RFfS, editor. Vienna, Austria 2012.

[67] Niehoff B, Schmithusen T, Knuppel N, Daase M, Czerny J, Boxhammer T. Mesozooplankton community development at elevated CO_2_ concentrations: results from a mesocosm experiment in an Arctic fjord. Biogeosciences. 2013;10(3):1391–406.

[68] Thor P, Oliva EO. Ocean acidification elicits different energetic responses in an Arctic and a boreal population of the copepod *Pseudocalanus acuspes*. Mar Biol. 2015;162(4):799–807.

[69] Thor P, Cervetto G, Besiktepe S, Ribera-Maycas E, Tang KW, Dam HG. Influence of two different green algal diets on specific dynamic action and incorporation of carbon into biochemical fractions in the copepod *Acartia tonsa*. J Plankton Res. 2002;24(4):293–300.

[70] Malzahn AM, Hantzsche F, Schoo KL, Boersma M, Aberle N. Differential effects of nutrient-limited primary production on primary, secondary or tertiary consumers. Oecologia. 2010;162(1):35–48. 10.1007/s00442-009-1458-y 19784675

[71] Taucher J, Haunost M, Boxhammer T, Bach LT, Algueró-Muñiz M, Riebesell U. Influence of ocean acidification on plankton community structure during a winter-to-summer succession: An imaging approach indicates that copepods can benefit from elevated CO_2_ via indirect food web effects. PLoS ONE. 2017;12(2):e0169737 10.1371/journal.pone.0169737 28178268 PMC5298333

[72] Bach LT, Boxhammer T, Larsen A, Hildebrandt N, Schulz KG, Riebesell U. Influence of plankton community structure on the sinking velocity of marine aggregates. Gobal Biogeochem Cy. 2016:n/a–n/a.

[73] Schoo KL, Malzahn AM, Krause E, Boersma M. Increased carbon dioxide availability alters phytoplankton stoichiometry and affects carbon cycling and growth of a marine planktonic herbivore. Mar Biol. 2013;160(8):2145–55.

[74] Niehoff B. Gonad morphology and oocyte development in Pseudocalanus spp. in relation to spawning activity. Mar Biol. 2003;143(4):759–68.

[75] Eberlein T, Wohlrab S, Rost B, John U, Bach LT, Riebesell U, et al Impacts of ocean acidification on primary production in a coastal North Sea phytoplankton community. PLoS One. 2017. 10.1371/journal.pone.0172594 PMC5342202 28273107

[76] Checkley DM. Selective feeding by Atlantic herring (*Clupea harengus*) larvae on zooplankton in natural assemblages. Marine Ecology—Progress Series. 1982;9:245–53.

[77] Hufnagl M, Peck MA. Physiological individual-based modelling of larval Atlantic herring (*Clupea harengus*) foraging and growth: insights on climate-driven life-history scheduling. ICES J Mar Sci. 2011;68(6):1170–88.

[78] Denis J, Vallet C, Courcot L, Lefebvre V, Caboche J, Antajan E, et al Feeding strategy of Downs herring larvae (*Clupea harengus* L.) in the English Channel and North Sea. J Sea Res. 2016;115:33–46.

[79] Cripps G, Lindeque P, Flynn KJ. Have we been underestimating the effects of ocean acidification in zooplankton? Global Change Biol. 2014;20:3377–85. 10.1111/gcb.12582 24782283

[80] Fitzer SC, Caldwell GS, Close AJ, Clare AS, Upstill-Goddard RC, Bentley MG. Ocean acidification induces multi-generational decline in copepod naupliar production with possible conflict for reproductive resource allocation. J Exp Mar Biol Ecol. 2012;418–419:30–6.

[81] Condon RH, Graham WM, Duarte CM, Pitt KA, Lucas CH, Haddock SHD, et al Questioning the rise of gelatinous zooplankton in the world's oceans. Bioscience. 2012;62(2):160–9.

[82] Purcell JE. Jellyfish and ctenophore blooms coincide with human proliferations and environmental perturbations. Annu Rev Mar Sci. 2012;4(1):209–35. 10.1146/annurev-marine-120709-142751 22457974

[83] Kikkawa T, Minowa Y, Nakamura Y, Kita J, Ishimatsu A. Swimming inhibition by elevated pCO_2_ in ephyrae of the scyphozoan jellyfish, *Aurelia*. Plankton Benthos Res. 2010;5(3):119–22.

[84] Algueró-Muñiz M, Meunier CL, Holst S, Alvarez-Fernandez S, Boersma M. Withstanding multiple stressors: ephyrae of the moon jellyfish (*Aurelia aurita*, Scyphozoa) in a high-temperature, high-CO_2_ and low-oxygen environment. Mar Biol. 2016;163(9):1–12.

[85] Richardson AJ, Gibbons MJ. Are jellyfish increasing in response to ocean acidification? Limnol Oceanogr. 2008;53(5):2040–5.

